# Entropically engineered formation of fivefold and icosahedral twinned clusters of colloidal shapes

**DOI:** 10.1038/s41467-022-34891-5

**Published:** 2022-11-30

**Authors:** Sangmin Lee, Sharon C. Glotzer

**Affiliations:** 1 Department of Chemical Engineering, University of Michigan, Ann Arbor, MI USA; 2 Biointerfaces Institute, University of Michigan, Ann Arbor, MI USA; 3 Present Address: Department of Biochemistry, University of Washington, Seattle, WA USA

**Keywords:** Nanoparticles, Colloids

## Abstract

Fivefold and icosahedral symmetries induced by multiply twinned crystal structures have been studied extensively for their role in influencing the shape of synthetic nanoparticles, and solution chemistry or geometric confinement are widely considered to be essential. Here we report the purely entropy-driven formation of fivefold and icosahedral twinned clusters of particles in molecular simulation without geometric confinement or chemistry. Hard truncated tetrahedra self-assemble into cubic or hexagonal diamond colloidal crystals depending on the amount of edge and vertex truncation. By engineering particle shape to achieve a negligible entropy difference between the two diamond phases, we show that the formation of the multiply twinned clusters is easily induced. The twinned clusters are entropically stabilized within a dense fluid by a strong fluid-crystal interfacial tension arising from strong entropic bonding. Our findings provide a strategy for engineering twinning behavior in colloidal systems with and without explicit bonding elements between particles.

## Abstract (web summary)

Fivefold and icosahedral symmetries in multiply twinned crystals can be used to influence the shape of synthetic nanoparticles. Simulations now show the entropy-driven formation of fivefold and icosahedral twinned clusters of truncated tetrahedra that self-assemble into colloidal crystals.

## Introduction

Twinning arises from a type of grain boundary called a twin boundary, where two separate crystals sharing the same lattice plane intergrow with a certain symmetry. Fivefold and icosahedral symmetries, which are generally known to be incompatible with long-range order, can be induced in relatively large atomic crystal clusters (from several nanometers to a few microns) via multiple twinning^1–4^. For instance, materials that form face-centered cubic (fcc)^2,3,5–7^ or cubic diamond^8,9^ crystals can form twin boundaries toward the (111) or its equivalent lattice directions, promoting the formation of a $\sim 70^\circ$ twin angle that can produce multiply twinned structures with fivefold or icosahedral symmetry. Twinning has been widely used to obtain interesting properties in synthetic nanomaterials, such as enhancing the mechanical properties of nanowires^10^ and increasing oxidation resistance^11^, and to synthesize noble metal nanostructures with decahedral or icosahedral shape^3,12^, which are useful as catalysts^13^. Many studies have been conducted to control the growth mechanism^12,14,15^ and stability of multiply twinned structures, e.g., tailoring solution chemistry^5,6,16–18^ and using multicomponent materials^6,11^. However, the multiplicity of variables in multiply twinned crystals makes it difficult to know which variables are responsible for, or have the most influence on, the formation of fivefold and icosahedral twins.

Compared to most systems, hard particle systems are regarded as relatively simple because the phase behavior is driven solely by entropy maximization^19,20^. The diversity and complexity of entropy-driven phase behavior, however, are enormously rich. The self-assembly of complex crystals and quasicrystals^20,21^, as well as complex assembly pathways such as multistep crystallization, have been reported in hard particle systems^22^. The spontaneous formation of icosahedral twins has been observed in hard sphere systems both in experiments and in computer simulations, but only under spherical confinement^4,7,15,23^, which produces an artificial “surface tension” to overcome the internal strain within the cluster arising from local particle packing. That, and the negligible free energy difference between fcc and hexagonal close-packed (hcp), two competing crystal structures of hard spheres^24^, conspire to produce twins. However, the formation and stabilization of fivefold and icosahedral twinned clusters without geometrical confinement or in any other hard particle systems or have yet to be reported. We hypothesized that artificial surface tension provided by spherical confinement could be achieved naturally, without any confinement, through the judicious choice of particle shape.

**Fig. 1:**
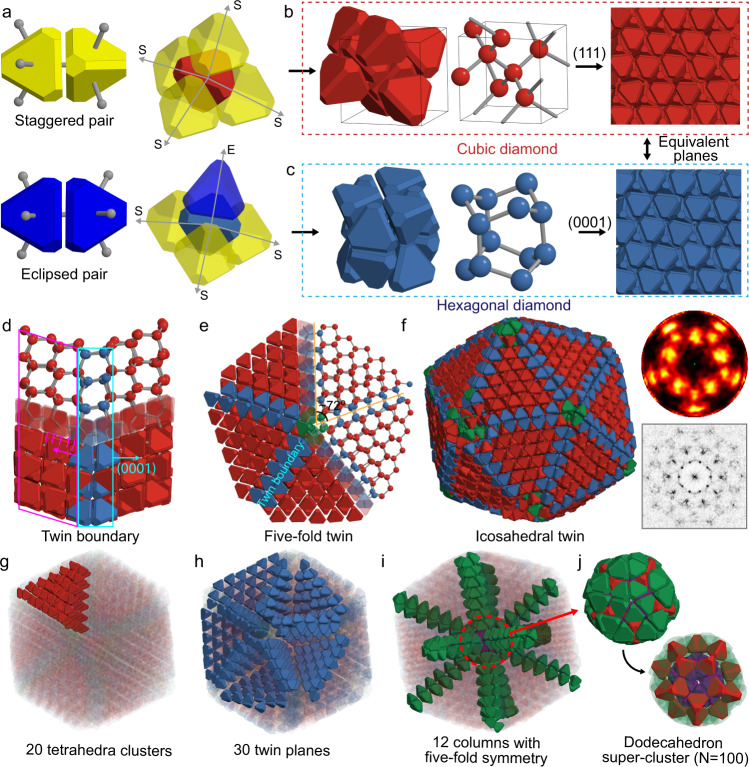
Twinned crystals of truncated tetrahedra. **a** Two types of pairwise contacts (left) and the local environment of particles in cubic diamond (upper right) and hexagonal diamond (bottom right). **b**, **c** Unit cells of cubic diamond (**b**, left and middle) and hexagonal diamond (**c**, left and middle) crystals. The (111) plane of cubic diamond (**b**, right) and the (0001) plane of hexagonal diamond (**c**, right) have the same structure. **d**–**j** Twinned structures of TTs. Red particles are ${{{{{{\rm{S}}}}}}}_{4}{{{{{{\rm{E}}}}}}}_{0}$, blue are ${{{{{{\rm{S}}}}}}}_{3}{{{{{{\rm{E}}}}}}}_{1}$, green are ${{{{{{\rm{S}}}}}}}_{2}{{{{{{\rm{E}}}}}}}_{2}$ and purple are ${{{{{{\rm{S}}}}}}}_{1}{{{{{{\rm{E}}}}}}}_{3}$. **d** A twin boundary is formed when the (111) plane of cubic diamond (magenta arrow) and the (0001) plane of hexagonal diamond (cyan arrow) meet. **e**, Five-fold twinned structure of TTs. The angle between twin boundaries is ~72^o^. **f**–**j** Structure of icosahedral twinned crystal. The bond orientational order diagram (**f**, upper right) and diffraction pattern (**f**, bottom right) along the 10-fold symmetry axis. The ico-twin crystal consists of 20 cubic diamond clusters with a tetrahedron Wulff shape (red in **f**, **g**), 30 twin planes between the tetrahedral clusters (blue in **f**, **h**), and 12 columns with 5-fold symmetry (green in **f**, **i**). **j** The center of the ico-twin is a dodecahedron super-cluster of 100 TTs.

**Fig. 2:**
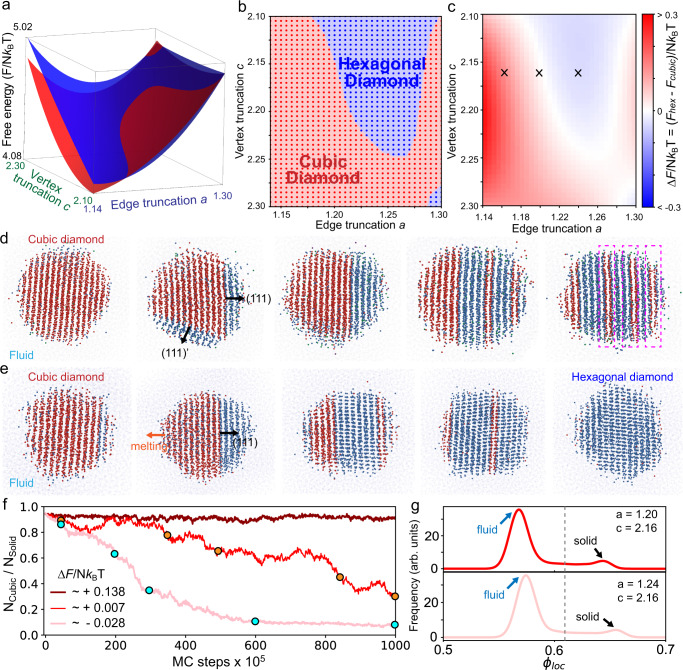
Stability control of diamond crystals via particle shape design. **a** Per-particle Helmholtz free-energy ($F/N{k}_{B}T$) plot, in units of *k*_*B*_*T*, of cubic (red) and hexagonal (blue) diamond crystals of hard TTs as a function of vertex and edge truncation parameters at constant particle volume fraction ($\phi=0.62$). **b** Phase diagram of hard TTs in the shape space determined by the free-energy calculation. **c** Free-energy difference between cubic and hexagonal diamond ($\triangle F={F}_{{{{{{\rm{H}}}}}}}-{F}_{{{{{{\rm{C}}}}}}}$) in shape space. **d**, When the TT is designed $(a=1.20,\,{c}=2.16)$ to have negligible $\triangle F \sim+0.007$, the initial cubic diamond (left) single-crystalline cluster forms multiple stacking faults (right) in fluid, suggesting that the free energy loss from the twin boundaries is small. Each TT is represented by a tiny sphere at the TT center of mass. Red and blue spher**e**s represent cubic and hexagonal diamonds, respectively. **e** When the TT is designed ($a=1.22,\,{c}=2.16$) to have $\triangle F \sim -0.028$, the initial cubic diamond cluster (left) completely transforms into single-crystalline hexagonal diamond (right) cluster in a relatively short simulation time. **f** The change of the number ratio of parti**c**les in cubic diamond over time for three different $\triangle F=+0.138,+0.007$ and $-0.028$ (black X markers in **c**). Orange and cyan circles indicate each snapshot in **d** and **e**, respectively. **g**, Local volume fraction distribution plots calculated from the last snapshot from **d** (upper) and **e** (lower). Two peaks in the distribution show that the system is in coexistence between fluid and solid.

**Fig. 3:**
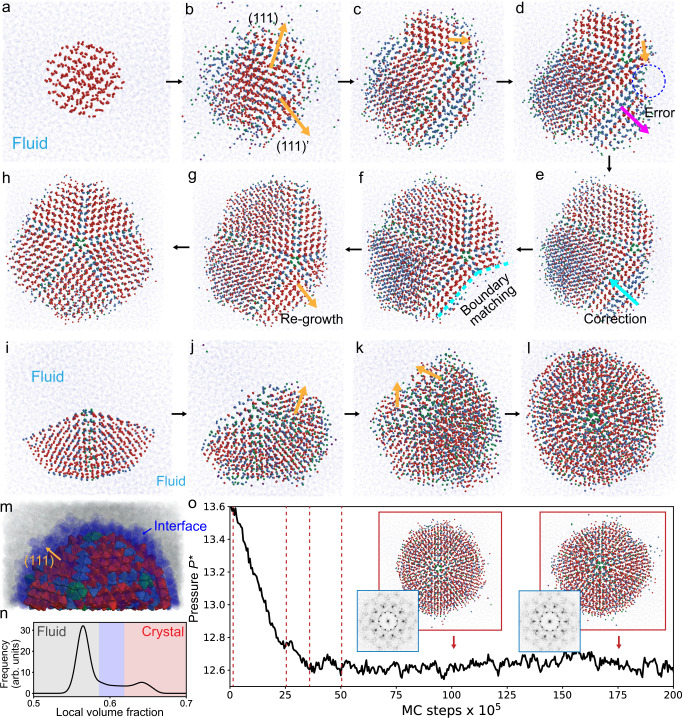
Growth process of fivefold and icosahedral twinned crystals from seed in fluid. Seed assisted growth of twinned crystals of hard TTs ($a=1.20,\,{c}=2.16$) at coexistence between crystal and fluid ($\phi=0.58$). **a**–**h** Simulation snapshots showing the error-and-repair mechanism of the five-fold twinned crystal. Each TT is represented by a tiny sphere at the TT center of mass. **a** When a spherical seed of cubic diamond crystal ($N=500$) grows, **b** twin boundaries are formed along (111) or its equivalent plane directions. Because the growth of each direction is independent, **c**, **d** an error can occur when stacking sequences of two growth directions mismatch. **e** The growing direction with the error is re-melted toward its opposite direction (**f**) until the boundary with its adjacent plane matches. Once the boundary matches, **g**, **h**) the five-fold twinned crystal grows to fully form the five-fold twinned crystal with truncated pentagonal dipyramid (PD) crystal shape. The final crystal is fully surrounded by fluid and stabilized. **i**–**l** Simulation snapshots of icosahedral twinned crystal formation from PD seed. All particles are represented by spheres showing centers of mass. **m** The icosahedral twinned crystal exposes (111) surfaces of cubic diamond crystal when stabilized in fluid. **n** Local volume fraction distribution plot of the system at coexistence shows a bimodal shape at $\phi=0.57$ and $0.64$. **o** The change of pressure over time for the ico-twin crystal-forming system. Pressure decreases during the growth of the ico-twin crystal and is constant (${P}^{*} \sim 12.6$) after the growth, indicating that the ico-twin crystal is stable in fluid. The red dashed lines indicate the simulation time when the snapshots in **i**–**l** are taken. The inset snapshots and diffraction patterns show that the ico-twin crystal structure surrounding by fluid is maintained during pressure stabilization.

**Fig. 4:**
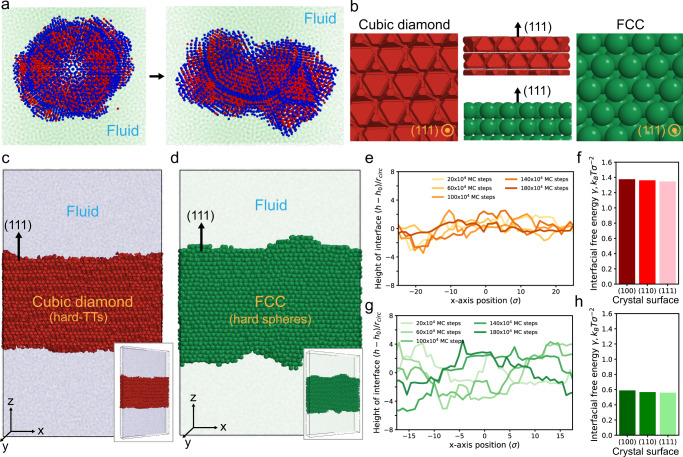
Fluid-solid interfacial energy calculation. **a** Icosahedral twinned crystal of FCC of hard spheres quickly destabilizes in fluid ($$ < {10}^{6}$$ MC steps). **b** (111) surfaces of a cubic diamond crystal of hard TTs (left) and an FCC crystal of hard spheres (right), and their side views (middle). Simulation setup of the capillary fluctuation method for calculating the fluid-solid interfacial stiffness $$\widetilde{\gamma }$$ of (**c**) cubic diamond and (**d**) FCC. **e**–**g** The change of interfacial profiles of the (111) direction of (**e**) cubic diamond and (**g**) FCC. **f**–**h** Fluid-solid interfacial free energy $$\gamma$$ of (**f**) cubic diamond and (**h**) FCC. For all three lattice directions, the hard TT system has more than twice the value of $$\gamma$$ of the hard sphere system, indicating its stronger fluid-solid surface tension.

Here, we show the purely entropy-driven, confinement-free assembly of fivefold and icosahedral twinned clusters from multiply twinned diamond crystals in equilibrium with a dense fluid phase. Monte Carlo (MC) simulations show that hard truncated tetrahedrons (TTs) self-assemble into either cubic and hexagonal diamond crystals depending on the amount of edge and vertex truncation (Fig. 1). We tuned the TT shape to have a negligible free energy difference between the two diamond crystal phases, and found the formation of twin boundaries is easily induced in a fluid phase (Fig. 2). This finding demonstrates that the stability of twin boundaries in a fluid can be controlled by particle shape design, even entropically. Using this strategy, we induce the formation of fivefold and icosahedral twins through seed-assisted growth of a single-crystalline seed of cubic diamond. Since the formation of twin boundaries is easily induced toward multiple directions during crystal growth, multiply twinned structures are easily formed. We show that, through an error-and-repair process, a multiply twinned structure with defects transforms into a fivefold twinned cluster in a dense fluid phase (Fig. 3). We also show the formation of an icosahedral twin in a dense fluid upon further growth of the fivefold twin. The icosahedral twin of hard TTs is entropically stabilized within the fluid without spherical confinement (Fig. 3), unlike the icosahedral cluster of hard spheres that rapidly destabilizes and falls apart (Fig. 4). We show that icosahedral clusters of hard TTs have twice the fluid-solid interfacial free-energy (or entropy) compared to icosahedral clusters of hard spheres as a natural consequence of stronger entropic bonding^25^ in the former system. Our study isolates the essential role of surface tension in stabilizing the inherently strained icosahedral twinned cluster in a dense fluid, and suggests approaches for engineering the surface tension.

## Results

Cubic diamond is different from hexagonal diamond (or lonsdaleite) in the conformation of local tetrahedral bonds^26^. Each atom has four tetrahedral bonds with nearest neighbors, and every atom in cubic diamond has four staggered bonds, while every atom in hexagonal diamond has three staggered bonds and one eclipsed bond. For a hard particle with a tetrahedron shape, the two types of chemical bond conformations can be mapped to the staggered and eclipsed entropic bond conformations associated with face-to-face contacts. (Fig. 1a). An appropriate degree of tip truncation of hard tetrahedra allows them to exclusively form staggered contacts, resulting in the self-assembly of the cubic diamond phase^27,28^ (Fig. 1b). However, the self-assembly of the hexagonal diamond phase, where the two bond conformation types are mixed, has not been reported in hard particle systems. We hypothesized that, through judicious truncation of a regular tetrahedron, we can find an appropriate shape that introduces both staggered and eclipsed contacts and favors hexagonal diamond (Fig. 1c), and, intermediate between the cubic diamond-forming and hexagonal diamond forming shapes, one that produces both diamond structures with negligible difference in entropy. Controlling the relative stability of cubic and hexagonal diamond will give us a route to control twinning and stacking behaviors of diamond crystals (Fig. 1b–d). Cubic and hexagonal diamond share an equivalent plane along the $(111)$ and $(0001)$ directions (Fig. 1b, c); fcc and hcp share the same equivalent plane. If the entropy difference between the two crystals can be engineered to be sufficiently small, the crystal will be prone to form twin boundaries.

The twinning of cubic diamond occurs towards $(111)$ and its equivalent planes such as $(\bar{1}11)$, $(1\bar{1}1)$ and $(11\bar{1})$; thus when twinning occurs in multiple directions, two twin planes meet with a specific angle, ${{{\cos }}}^{-1}(1/3)=70.5^\circ$, which is close to $72^\circ$, the same angle that promotes the formation of multiply twinned structures with fivefold symmetry^2^ (Fig. 1e). The structure of the multiply twinned crystals made by TTs is easily analyzed by classifying particles based on the number of staggered and eclipsed contacts (Supplementary Figs. 1, 2), following the notation ${{{{{{\rm{S}}}}}}}_{n}{{{{{{\rm{E}}}}}}}_{m}$, where $n+m=4$. For instance, the cubic diamond crystal consists of ${{{{{{\rm{S}}}}}}}_{4}{{{{{{\rm{E}}}}}}}_{0}$ particles (Fig. 1a, b), and the hexagonal diamond crystal consists of ${{{{{{\rm{S}}}}}}}_{3}{{{{{{\rm{E}}}}}}}_{1}$ particles (Fig. 1a, c). In a fivefold twin, five cubic diamond clusters with a tetrahedron shape made by ${{{{{{\rm{S}}}}}}}_{4}{{{{{{\rm{E}}}}}}}_{0}$ particles together form a pentagonal bipyramid super-cluster with five twin planes of ${{{{{{\rm{S}}}}}}}_{3}{{{{{{\rm{E}}}}}}}_{1}$ particles between the faces of the tetrahedron clusters (Fig. 1e). At the point where the five twin planes meet, ${{{{{{\rm{S}}}}}}}_{2}{{{{{{\rm{E}}}}}}}_{2}$ particles form a column where small pentagonal bipyramids ($N=5$) are linearly stacked. An icosahedral twin (Fig. 1f) comprises twenty cubic diamond clusters (${{{{{{\rm{S}}}}}}}_{4}{{{{{{\rm{E}}}}}}}_{0}$ particles), each with a tetrahedron shape (Fig. 1g). Thirty twin planes (${{{{{{\rm{S}}}}}}}_{3}{{{{{{\rm{E}}}}}}}_{1}$) exist between the faces of the tetrahedron clusters (Fig. 1h), and there twelve fivefold columns made by ${{{{{{\rm{S}}}}}}}_{2}{{{{{{\rm{E}}}}}}}_{2}$ particles where five tetrahedron clusters meet (Fig. 1i). At the center, there is a dodecahedron super-cluster $(N=100)$ comprising three different shells with icosahedral symmetry: 60 ${{{{{{\rm{S}}}}}}}_{2}{{{{{{\rm{E}}}}}}}_{2}$ particles comprising the outer shell form a rhombicosidodecahedron, 20 ${{{{{{\rm{S}}}}}}}_{4}{{{{{{\rm{E}}}}}}}_{0}$ particles comprising the intermediate shell form a dodecahedron and 20 ${{{{{{\rm{S}}}}}}}_{1}{{{{{{\rm{E}}}}}}}_{3}$ particles comprising the smallest shell form a dodecahedron. This hierarchical structure is equivalent to the super-cluster of water ${({{{{{{\rm{H}}}}}}}_{2}{{{{{\rm{O}}}}}})}_{100}$ when connecting oxygen atoms^29^.

A competition between staggered and eclipsed contracts arises from many-body interactions responsible for the entropic forces producing the effective attraction between neighboring particles, and preferences to form a certain type of face-to-face TT contact—which are determined by the strength of this attraction—can be controlled by varying the amount of edge and vertex truncation^28^. We know that regular tetrahedra prefer to form a dodecagonal quasicrystal^21^ in which every particle has eclipsed alignments (${{{{{{\rm{S}}}}}}}_{0}{{{{{{\rm{E}}}}}}}_{4}$). Truncation introduces additional facets, weakening the strength of the entropic bond between two primary eclipsed faces, and thereby weakening the preference for eclipsed alignment. The entropic bonding between primary eclipsed faces is weakened with increasing truncation because less and less free volume is gained by maintaining this alignment (though this alignment still maximizes free volume and thus system entropy). Beyond a certain amount of truncation, however, the system has more entropy (more free volume) if the neighbors align in a staggered way, and thus the staggered configuration becomes preferred over the eclipsed configuration (${{{{{{\rm{S}}}}}}}_{4}{{{{{{\rm{E}}}}}}}_{0}$)^27^. Motivated by this, we searched the shape space^28^ between ${{{{{{\rm{S}}}}}}}_{0}{{{{{{\rm{E}}}}}}}_{4}$ and ${{{{{{\rm{S}}}}}}}_{4}{{{{{{\rm{E}}}}}}}_{0}$ to find a shape intermediate between the two that favors the hexagonal diamond phase $({{{{{{\rm{S}}}}}}}_{3}{{{{{{\rm{E}}}}}}}_{1})$. To quantify the relative thermodynamic stability of cubic and hexagonal diamond as a function of TT shape, we constructed free energy surfaces in TT shape space varying the edge ($1.14\le a\le 1.30$) and vertex ($2.10\le c\le 2.30$) truncation at constant volume fraction (Fig. 2a), using the Frenkel–Ladd free energy calculation method^24,30^ (“Methods”). We found a region where hexagonal diamond is thermodynamically more stable than cubic diamond, as shown in the phase diagram generated from the bottom view of the free energy landscape (Fig. 2b). We also confirmed those shapes self-assemble hexagonal diamond (Supplementary Fig. 3).

To assess the stability of the twin boundaries depending on the TT shape, we chose three systems with different relative stabilities between the two diamond phases, $\Delta F={F}_{H}-{F}_{C}=0.138,0.007,\,{{{{{\rm{and}}}}}}-0.028$ (Fig. 2c). For each system, we prepared a cubic diamond cluster fully surrounded by a fluid phase at coexistence and equilibrated the system (“Methods”). We first distinguished particles in fluid and crystal based on the local volume fraction (“Methods”), which shows two peaks in distribution plots that indicate the two coexisting phases (Fig. 2g). Then, we colored particles in the crystal phase based on their face-to-face contact type $({{{{{{\rm{S}}}}}}}_{n}{{{{{{\rm{E}}}}}}}_{m})$ and analyzed the crystal structures. When the free energy difference is large enough $\left(\Delta F \sim+0.138\right)$, the cubic diamond cluster is stable without any notable changes in the crystal structure (Fig. 2f). However, when $\Delta F$ is negligible ($\Delta F \sim 0.007$), the initial cubic diamond cluster repeatedly developed stacking faults (Fig. 2d) during the simulation, rather than stabilizing either diamond phase. This shows that the formation of twin boundaries between the two diamond phases is easily induced when the thermodynamic stability of the two crystals is comparable. On the other hand, when we further increased the stability of hexagonal diamond ($\Delta F=-\!\!0.028$), a complete phase transformation into a single-crystalline hexagonal diamond was observed (Fig. 2e). The phase transition behavior of the three systems was quantitatively compared by the change of the number ratio of ${{{{{{\rm{S}}}}}}}_{4}{{{{{{\rm{E}}}}}}}_{0}$ particles in a solid phase (${N}_{{{{{{\rm{cubic}}}}}}}/{N}_{{{{{{\rm{solid}}}}}}}$) (Fig. 2f). We confirmed a more gradual decrease of the ${N}_{{{{{{\rm{cubic}}}}}}}/{N}_{{{{{{\rm{solid}}}}}}}$ in the $\Delta F=0.007$ system compared to that of the $\Delta F=-\!\!0.028$ system, due to frequent formation of stacking faults.

Based on these findings, we hypothesized that the formation of a multiply twinned structure might be easily induced during the growth of the cubic diamond when $\Delta F$ is negligible because of the easy formation of twin boundaries in multiple directions, which kinetically promotes the formation of ~$72^\circ$ twin angles. To confirm the hypothesis, we conducted seed-assisted growth simulations (“Methods”) with a system $(a=1.20,\,{c}=2.16)$ where $\Delta F$ is negligible $(\Delta F=0.007)$. We placed a small spherical seed of cubic diamond crystal ($N=500$) in a fluid and allowed the seed to grow at the coexistence volume fraction $\phi=0.58$ where the resulting crystal cluster is fully surrounded by a fluid phase in equilibrium. In the early stage of growth, we observed the formation of twin boundaries in multiple directions (Fig. 3a, b). Through subsequent multiple twinning events, a fivefold center was formed (Fig. 3c). Although the fivefold centers form a new local structure, ${{{{{{\rm{S}}}}}}}_{2}{{{{{{\rm{E}}}}}}}_{2}$ (Supplementary Fig. 2)—a pentagonal column —it has a similar local structure to that of hexagonal diamond, resulting in minimal entropy loss. Note that an error or defect can easily occur during the growth process because the formation of twin boundaries independently in multiple directions can cause a mismatch in the stacking sequence between two adjacent directions (Fig. 3d). However, interestingly, we observed a self-repair process during growth, where a growing direction with stacking errors re-melts until the boundary with its adjacent plane matches (Fig. 3e, f). Once the boundary matches, the fivefold twin crystal fully grows and is stabilized in fluid with a truncated pentagonal dipyramid shape (Fig. 3g, h). This error-and-repair mechanism of fivefold twinned crystals occurs via particle migration on the crystal surface, differently from the error-and-repair mechanism recently reported, in which particle rearrangement occurs throughout the fivefold twinned crystal during growth^14^, and differently from that reported recently for a dodecagonal quasicrystal of hard tetrahedra^31^. The observation of this new mechanism demonstrates that there exist multiple types of error-and-repair mechanisms for the formation of fivefold twinned clusters as well as for entropically stabilized colloidal crystals.

Next, we investigated the formation of an icosahedral twinned cluster within a fluid phase. Experiments have shown that an icosahedral twinned cluster can be formed from additional multiple twinning from a decahedral cluster^12,32^. Simulations have shown it can be formed in a purely entropy-driven system provided there is artificial confinement of the cluster. Our simulation results show that this is possible also in a purely entropy-driven system, but without confinement. We conducted a growth simulation of a seed with a fivefold twinned structure $(N=1020)$ in a system where $\Delta F$ is negligible ($\Delta F \sim 0.007$ at $a=1.20,\,{c}=2.16$) (“Methods”), and where the solid cluster is fully surrounded by fluid throughout the simulation. We observed the additional twinning from the surface of the seed, which eventually grew into an icosahedral twin (Fig. 3i–l), a process reminiscent of a simultaneous successive twinning of a palladium icosahedral cluster^32^. The fluid-solid coexistence of our ico-twin was confirmed by the local volume fraction distribution (Fig. 3n) showing two peaks. We classified particles into three types: fluid, interface and crystal. After removing particles at the interface, we confirmed that the icosahedral cluster exposes twenty (111) planes of the cubic diamond crystal toward the fluid (Fig. 3m). This implies that the interfacial entropy difference between fluid and the (111) plane plays an important role in the stability of the icosahedral cluster^17^.

The stability of the icosahedral cluster in the fluid can be checked by tracking the change in reduced system pressure (${P}^{*}=P{v}_{0}/{k}_{B}T$) during a simulation run (Fig. 3o). In the early stage ($\le 50\times {10}^{5}$ MC steps), we observed a rapid decrease in pressure due to the growth of the icosahedral cluster. After the growth ($> 50\times {10}^{5}$ MC steps), the pressure stabilized at a constant value (${P}^{*} \sim 12.6$) with small fluctuations, indicating that the ico-twin structure had stabilized in the fluid. From the simulation snapshots and diffraction patterns at $100\times {10}^{5}$ and $175\times {10}^{5}$ MC steps, we confirmed that the icosahedral symmetry of the solid cluster was maintained (Fig. 3o, insets).

Structurally, an icosahedral twin has internal and surface strain^33^, thus an internal or external effect to compensate for the strain energy is required to stabilize the cluster. For instance, we know that hard sphere systems require spherical confinement to entropically stabilize an icosahedral cluster^4,7,23^, otherwise, the icosahedral cluster rapidly destabilizes (Fig. 4a). This indicates that the fluid-solid interfacial tension of hard spheres is not strong enough to overcome the entropy loss from the strain within the icosahedral cluster. On the other hand, the icosahedral cluster of hard TTs is stable in a fluid without geometric confinement, suggesting that the fluid-solid interfacial tension is enhanced by the shape of the particle. Figure 4b shows the crystal structure of the (111) plane in the clusters of hard TTs and hard spheres, which is the largest surface of the icosahedral cluster exposed to the fluid. The (111) surface of the hard TT crystal is flat because the faces of the TTs are exposed toward the plane. On the other hand, the (111) surface of the hard sphere crystal is rough due to the spherical shape of the particles, implying that the roughness of the surface can affect the surface tension of the crystal.

To quantify the difference in the surface effect, we calculated the fluid-solid interfacial free energy of hard TTs and hard spheres using the capillary fluctuation method^34^ (“Methods”). We exposed a specific lattice plane of a crystal toward a fluid in a thin slab ($z$-axis in Fig. 4c, d) and monitored the fluctuation of the interface in equilibrium. The interfacial profile normalized by the circumscribed radius of the particle, $(h-{h}_{0})/{r}_{{{{{{\rm{circ}}}}}}}$, shows that the fluctuations of the (111) plane of the hard sphere system are larger than that of the hard truncated tetrahedra system (Fig. 4e and Supplementary Movies 1, 2). From 200 samples of the interfacial profiles obtained in equilibrium, we calculated the interfacial stiffness ($\widetilde{\gamma }$) of the crystal planes (Supplementary Fig. 6 and “Methods”) and measured the fluid-solid interfacial free energy $(\gamma )$ of several major crystal orientations (Supplementary Table 2). The interfacial stiffness of the (111) plane of the fcc crystal of hard spheres, ${\widetilde{\gamma }}_{{{{{{\rm{HS}}}}}\_}(111)}=0.684{k}_{B}T{\sigma }^{-2}$, is $56\%$ lower than that of the cubic diamond crystal of hard truncated tetrahedra, ${\widetilde{\gamma }}_{{{{{{\rm{TT}}}}}\_}(111)}=1.547{k}_{B}T{\sigma }^{-2}$ (“Methods”). In addition, we confirmed that the fluid-solid interfacial free energy difference (surface tension) of the (111) plane of the hard TT crystal, ${\gamma }_{{{{{{\rm{TT}}}}}\_}(111)}=1.347{k}_{B}T{\sigma }^{-2}$, is $\sim 2.4$ times higher than that of the hard sphere crystal, ${\gamma }_{{{{{{\rm{HS}}}}}\_}(111)}=0.560{k}_{B}T{\sigma }^{-2}$ (Supplementary Table 2). This indicates that a strong fluid-solid interfacial tension of the hard TT system stabilizes the icosahedral twinned cluster in fluid without geometric confinement.

The dependence of the fluid-solid interfacial tension on particle shape is indirectly indicated by the change in local volume fraction distributions arising from TT shape change (Supplementary Fig. 5). The volume fraction of fluid and crystal at fluid-solid coexistence depends on the fluid-solid surface tension; thus, the local volume fraction change due to TT truncation suggests a change in fluid-solid interfacial energy from the TT shape change. To confirm this hypothesis, we compared three different hard particle systems with very different local volume fraction distributions at fluid-solid coexistence (Supplementary Fig. 7): (1) Hard triangular bipyramids (TBP) forming clathrate type-1 crystal^22^, (2) hard TTs with $a=1.20$, $c=2.16$ forming cubic diamond, and (3) hard spheres (HS) forming FCC crystal. The three systems show different local volume fraction difference between crystal and fluid (${\phi }_{{{{{{\rm{Crystal}}}}}}}-{\phi }_{{{{{{\rm{Fluid}}}}}}}=\triangle \phi$): $\triangle {\phi }_{{{{{{\rm{TBP}}}}}}}=0.116$, $\triangle {\phi }_{{{{{{\rm{TT}}}}}}}=0.077$ and $\triangle {\phi }_{{{{{{\rm{HS}}}}}}}=0.05$ (Supplementary Fig. 7). Next, we conducted capillary fluctuation simulations for the three systems to calculate the interfacial stiffness of each system. We observed that the TBPs with the largest local volume fraction difference ($\triangle \phi=0.116$) show the smallest fluctuations of the interface, resulting in the strongest interfacial stiffness ($\widetilde{\gamma } \sim 7.21{k}_{B}T{\sigma }^{-2}$) (Supplementary Fig. 7b, c). On the other hand, the HSs with the smallest local volume fraction difference ($\triangle \phi=0.05$) show the largest fluctuations of the interface, resulting in the weakest interfacial stiffness ($\widetilde{\gamma } \sim 0.74{k}_{B}T{\sigma }^{-2}$) (Supplementary Fig. 7h, i). This comparison provides clear evidence that particle shape determines the volume fraction of fluid and solid at coexistence, which in turn determines the interfacial energy.

In summary, we studied the entropy-driven assembly and stabilization of fivefold and icosahedral twinned clusters in a one-component fluid of hard truncated tetrahedra (TT). We showed that the thermodynamic stability of the cubic and hexagonal diamond phases can be entropically controlled by designing the TT shape. If the particle shape is designed to have a negligible free energy difference between the two diamond crystals, the formation of twin boundaries is easily induced. This strategy can be used to induce the formation of fivefold and icosahedral twinned clusters in fluid via seed-assisted growth. We found that the formation of a fivefold cluster follows an error-and-repair mechanism that removes mismatches of the stacking sequence of adjacent twin planes through particle rearrangement at the cluster surface. We showed the formation of an icosahedral cluster by additional growth of twinned structures from a fivefold twinned seed. We showed that the icosahedral cluster of TTs can be entropically stabilized in a fluid, which is not possible for hard spheres without confinement. The capillary fluctuation method showed that a hard TT system has a much higher fluid-solid interfacial free energy (here, entropy) difference than that of a hard sphere system, directly implicating fluid-solid interfacial tension in stabilizing the TT ico-twin.

Our findings provide a quantitative understanding of the formation and stabilization of fivefold and icosahedral twinned clusters in hard particle systems. Importantly, we showed that the twinning behavior and the interfacial free energy difference may be entropically engineered by particle shape design. Experimentally, colloidal tetrahedral particles are synthesizable^26,35–38^ with tunable shape^37^. Non-complementary DNA could be used to make non-interacting particles that might closely approximate those studied here. Fivefold and icosahedral twinned clusters should be also attainable with attractive TT particle shapes, provided the explicit attraction favors face-to-face alignment. Such interparticle attraction could be realized through organic ligands^36,38^ via van der Waals forces or self-complementary DNA^26^, both of which are commonly used to drive nanoparticle assembly.

## Methods

### Particle geometry

The truncated tetrahedron (TT) shape is a member of the spherical triangle invariant 323 family, with truncation parameters $\left(a,b,c\right)$ according to previous convention^39^. In this study, $b=1.0$ for all systems, and $a$ and $b$ vary in a range: $1.14\le a\le 1.30$ and $2.10\le c\le 2.30$.

### Identification of staggered and eclipsed pairs

Each TT in a crystal phase has four nearest neighbors that form face-to-face contacts. We identified the type of each face-to-face contact (staggered or eclipsed), following a similar identification protocol described in ref. 28. Briefly, for each face of the particle, we assigned three vectors that are defined from the face center to each tip of the face (Supplementary Fig. 1a). Then, for each pair contact ($i,j$), we can define three vectors for $i$ particle and three other vectors for $j$ particle (Supplementary Fig. 1b, c). We calculated every pair combination between the $i$ and $j$ vectors, which is nine, and found the minimum angle ($\theta$). All the $\theta$ from every pair contact accumulated from the entire system give an angle distribution. For instance, Supplementary Fig. 1d was calculated from hexagonal diamond crystal. The angle distribution shows a clear bimodal distribution, and the height of the peak at ${\theta }_{1}$ and ${\theta }_{2}$ are around a 1:3 ratio, indicating that the angles around ${\theta }_{1}$ and ${\theta }_{2}$ come from eclipsed pairs and staggered pairs, respectively. This allows us to select the range of the pair contact angle to identify the eclipsed pair (${\theta }_{1}\pm 10^\circ$) and the staggered pair (${\theta }_{2}\pm 10^\circ$).

### Monte Carlo simulation

Simulations were performed by the hard particle Monte Carlo (HPMC) implemented in the HOOMD-blue simulation package^40,41^, which is available at https://github.com/glotzerlab/hoomd-blue. The system size for the self-assembly simulations (Fig. 2) is $N={{{{\mathrm{2000}}}}}$, and all simulations were performed under periodic box condition. The translational and rotational movement of particles is decided to have around a $20\%$ acceptance ratio for every simulation. The self-assembly simulations were initialized from a dilute isotropic fluid $\phi$ $=N{v}_{0}/V < 0.01$ and compressed until the desired thermodynamic condition ($\phi$ or reduced pressure ${P}^{*}=P{v}_{0}/{k}_{B}T$) was reached. Here, ${v}_{0}$ and $V$ are the volume of a particle and the volume of the simulation box, respectively. The unit length of simulation is defined by $\sigma$, and the particle volume of a TT is set to ${{v}_{0}=1.0\sigma }^{3}$ regardless of its truncation amount. After initialization, each run was continued in the isochoric ensemble (NVT) at constant particle volume fraction $\phi$ until equilibration was reached. For the most systems, crystallization occurs within $1.0\times {10}^{8}$ Monte Carlo steps.

### Stability of diamond crystals in a dense fluid

We checked the stability of a cubic diamond crystal in a dense fluid for three different systems: (1) $a=1.16,\,{c}=2.16$ at $\phi=0.57$, (2) $a=1.20,\,{c}=2.16$ at $\phi=0.57$5 (Fig. 2d), and (3) $a=1.24,\,{c}=2.16$ at $\phi=0.58$ (Fig. 2e). All three systems are in a fluid-solid coexistence state, where a single-crystalline cluster is fully surrounded by a dense fluid phase. System size is $N={{{{\mathrm{20,000}}}}}$ for all three systems. For the MC simulation, we first placed a spherical cubic diamond single-crystalline cluster ($N \sim {{{{\mathrm{8000}}}}}$) at the center of a large simulation box $(\phi=0.01)$ and put other particles around the solid cluster without overlapping each other. Then, holding the particles of the crystal phase (no movements), we rapidly compressed the system to the target particle volume fraction. Then, we released the particles of the crystal and equilibrated the whole system.

### Free-energy calculation

The reduced Helmholtz free energy per particle $F/N{k}_{B}T$ of the cubic and hexagonal diamond phases were calculated using the Frenkel–Ladd method^24,30^ at a constant volume. The details of the calculation method can be found in ref. 30. Briefly, we first constructed an Einstein lattice (i.e., reference state) of each phase, for which the free energy is analytically solvable. Then, we applied strong translational and rotational harmonic potentials between the hard TTs and the Einstein lattice to tether each particle to its reference lattice site, which makes the hard TT system a reference state. Then we gradually released the harmonic potential until the strength of the potential became nearly zero, yielding the real system without correlation to the reference state. This process allows us to set up a continuous and reversible path between the reference state and a real state. Thus, by integrating the potential energy along the path, we can calculate the free energy (or equivalently, entropy) difference between the reference state and the real system. Because that the free energy of the reference crystal is analytically solvable, we can calculate the absolute free energy of the real crystal.

Using this method, we calculated the free energy of each phase with different shape parameters $(a,{c})$ to construct a free energy surface (Fig. 2a) in the shape space: $1.14\le a\le 1.30$ with 0.02 interval and $2.10\le c\le 2.30$ with 0.02 interval, for a total of 99 systems with different shape parameters $(a,{c})$. The system size is $N=512$ for the cubic diamond and $N=992$ for the hexagonal diamond. For each phase, we calculated the free energy of the 99 systems at $\phi=0.64$, $0.66$, $0.68$, $0.70$ and $0.72$, and for each $\phi$, the free energy surface was constructed by interpolating the 99 data points (Supplementary Fig. 4). The free energy of the crystal at $\phi=0.62$ (Fig. 2a) was estimated by extrapolating the free energy surface plots of the five particle volume fractions studied.

### Seed-assisted growth MC simulation

We conducted seed-assisted growth simulation at a fluid-solid coexistence state ($\phi=0.58$ for $a=1.20,\,{c}=2.16$ system). For the growth of the fivefold twinned cluster, we used a spherical cluster of a cubic diamond crystal ($N=500$) as a seed, and the total system size including the seed is $N={{{{\mathrm{20,000}}}}}$. At a very dilute condition ($\phi=0.01$), we placed the seed at the center of simulation box and placed other particles around the seed without overlap. Fixing the seed particles in place, we compressed the simulation box to the target particle volume fraction. Once it reached the target particle volume fraction, we released the seed particles and equilibrated the system. For the growth of the icosahedral twinned cluster, we used a fivefold twinned cluster ($N \sim {{{{\mathrm{1000}}}}}$) as a seed, and the total system size including the seed is $N={{{{\mathrm{20,000}}}}}$. The compression and equilibration protocol is the same in both cases.

### Local volume fraction calculation

Seed-assisted growth simulations for fivefold and icosahedral twinned clusters were performed in at coexistence between a fluid phase and a crystal phase, where the solid clusters are fully surrounded by fluid. To distinguish the two phases, we calculated the local particle volume fraction ${\phi }_{{{{{{\rm{loc}}}}}}}$, which is defined as the particle volume fraction around a particle within a certain radius, ${r}_{{{{{{{\rm{cut}}}}}}}}$. The value of ${r}_{{{{{{{\rm{cut}}}}}}}}$ in this study was taken to be 3 times the distance from a particle center to its nearest neighbor particle center, in order to properly average the local environment. TTs are identified as belonging to the fluid region if ${\phi }_{{{{{{\rm{loc}}}}}}}\le 0.60$ and to the crystal region if ${\phi }_{{{{{{\rm{loc}}}}}}} > 0.60$. We utilized the freud python library for this calculation^42^.

### Stability check of an icosahedral twinned cluster of hard spheres

We performed MC simulations of a hard sphere system to check the stability of an icosahedral twinned cluster of hard spheres at fluid-solid coexistence ($\phi=0.515$). We first constructed an icosahedral cluster ($N=5971$) with an ideal structure, following the method described in ref. 4. At a very dilute condition ($\phi=0.01$), we placed the ico-twin cluster at the center of simulation box and placed other hard spheres around the cluster without overlap. Temporarily “freezing” the ico-twin particles in place, we compressed the simulation box to the target particle volume fraction. Once it reached the target particle volume fraction, we released the ico-twin cluster particles and equilibrated the system. To check if the ico-twin cluster is stable in the coexistence phase, we monitored the bond-order diagram and the morphology of the ico-twin cluster during the simulation. We observed the icosahedral structure rapidly destabilize within $5\times {10}^{5}$ MC steps (Fig. 4a).

### Capillary fluctuation method

The fluid-solid interfacial free energy of hard TT ($a=1.20,\,{c}=2.16$) and hard sphere systems was calculated by the capillary fluctuation method^34^. We prepared systems in a fluid-solid coexistence state ($\phi=0.582$ for hard TTs and $\phi=0.515$ for hard spheres) containing a crystal, where the crystal of each system exposes different orientations toward a fluid phase: $(111)[\bar{1}10]$, $(100)[001]$, $(100)[\bar{1}10]$ and $(110)[001]$. Here, the crystal orientation is defined as $({ijk})[{lmn}]$, where $({ijk})$ is a crystal plane toward the fluid phase ($z$-direction in Fig. 4c, d) and $[{lmn}]$ is a crystal plane perpendicular to the fluid phase along the short direction ($y$-direction in Fig. 4c, d). The system size is $N \sim {{{{\mathrm{40,000}}}}}$ for every system, and the shortest direction of the simulation box contains 2–3 unit cells ($y$-direction). At coexistence, we distinguished fluid and solid phases using the local volume fraction ${\phi }_{{{{{{\rm{loc}}}}}}}$ for the hard TT system (${\phi }_{{{{{{\rm{loc}}}}}}} > 0.6$ for crystal phase) and the average bond-orientational order parameter^42,43^ ${\bar{q}}_{6}$ for the hard sphere system (${\bar{q}}_{6} > 0.3$ for crystal phase).

For the calculation of the fluid-solid interfacial free energy, we followed the calculation process described in ref. 34. Briefly, we obtained the interfacial profile $h(x)$ as a function of MC steps (Fig. 4e–g), which is then normalized by the circumscribed sphere radius of a particle. The values of $h(x)$ were measured at a discrete set of points ${x}_{n}=n\Delta$, where $n=1,\ldots,{N}$ and $\Delta={{L}}/{{N}}$, where *L* is the length of crystal along $x$-direction. The Fourier modes ${h}_{q}$ are defined as ${h}_{q}=\frac{1}{N}{\sum }_{n=1}^{N}h({x}_{n}){{{{{{\rm{e}}}}}}}^{{{{{{{\rm{i}}}}}}q}{x}_{n}}$, where wave number $q=\frac{2\pi k}{L}$ with $k=1,\ldots,{N}$. From the equipartition theorem, the size of the capillary fluctuation modes is related to the interfacial stiffness $\widetilde{\gamma }$ as follows^44^:

1 $$\left\langle {\left|{h}_{q}\right|}^{2}\right\rangle={k}_{B}T/A\widetilde{\gamma }{q}^{2}$$

where $A$ is the area of the fluid-crystal interface. The $\widetilde{\gamma }$ for different crystal orientations were calculated and averaged for various $q$ and $\varDelta x$ (Supplementary Fig. 6). The interfacial stiffness is related to the interfacial free energy $\gamma$ by the formula:

2 $$\widetilde{\gamma }\left(\theta \right)=\gamma \left(\theta \right)+\frac{{{{{{{\rm{d}}}}}}}^{2}\gamma }{{{{{{\rm{d}}}}}}{\theta }^{2}}$$

where $\theta$ is the angle between the average orientation of the interface and the local normal of the interface. Based on this relationship, the interfacial free energy can be obtained by comparing the following two equations:

3 $$\gamma \left({{{{{\boldsymbol{n}}}}}}\right)/{\gamma }_{0}=1+{A}_{1}{\epsilon }_{1}+{A}_{2}{\epsilon }_{2}$$

4 $$\widetilde{\gamma }\left({{{{{\boldsymbol{n}}}}}}\right)/{\gamma }_{0}=1+{B}_{1}{\epsilon }_{1}+{B}_{2}{\epsilon }_{2}$$

where ${{{{{\boldsymbol{n}}}}}}$ is the unit vector normal to the interfacial plane. The values of ${A}_{1}$, ${A}_{2}$, ${B}_{1}$ and ${B}_{2}$ for each interface direction is listed in Supplementary Table 1. Then, the interfacial free energy $\gamma$ for each crystal plane was obtained using the coefficients (Supplementary Table 1) and the interfacial stiffness (Supplementary Fig. 6). The calculation results are listed in Supplementary Table 2.

## Supplementary information

Supplementary Information

Description of Additional Supplementary Files

Supplementary Movie 1

Supplementary Movie 2

## Data Availability

All data needed to evaluate the conclusions in this manuscript are present in the main text or the supplementary materials. Additional data are available upon request to the corresponding author.

## Code availability

Source code for HOOMD-Blue is available at https://github.com/glotzerlab/hoomd-blue.
