# Supplementary material for: Entropically engineered formation of fivefold and icosahedral twinned clusters of colloidal shapes
Source: Nat Commun. 2022 Nov 30;13:7362. doi: 10.1038/s41467-022-34891-5 (PMC9712591; doi:10.1038/s41467-022-34891-5)
Supplement: Supplementary file 1 — Supplementary Information [file 41467_2022_34891_MOESM1_ESM.pdf]

Supplementary Information for

Entropically Engineered Formation of Fivefold and Icosahedral Twinned Clusters of Colloidal Shapes

Sangmin Lee[1,2] and Sharon C. Glotzer[1,3*]

[1]*Department of Chemical Engineering, University of Michigan, Ann Arbor, MI, USA.*

[2]*Present address: Department of Biochemistry, University of Washington, Seattle, WA, USA.*

[3]*Biointerfaces Institute, University of Michigan, Ann Arbor, MI, USA.*

**Correspondence to: sglotzer@umich.edu*

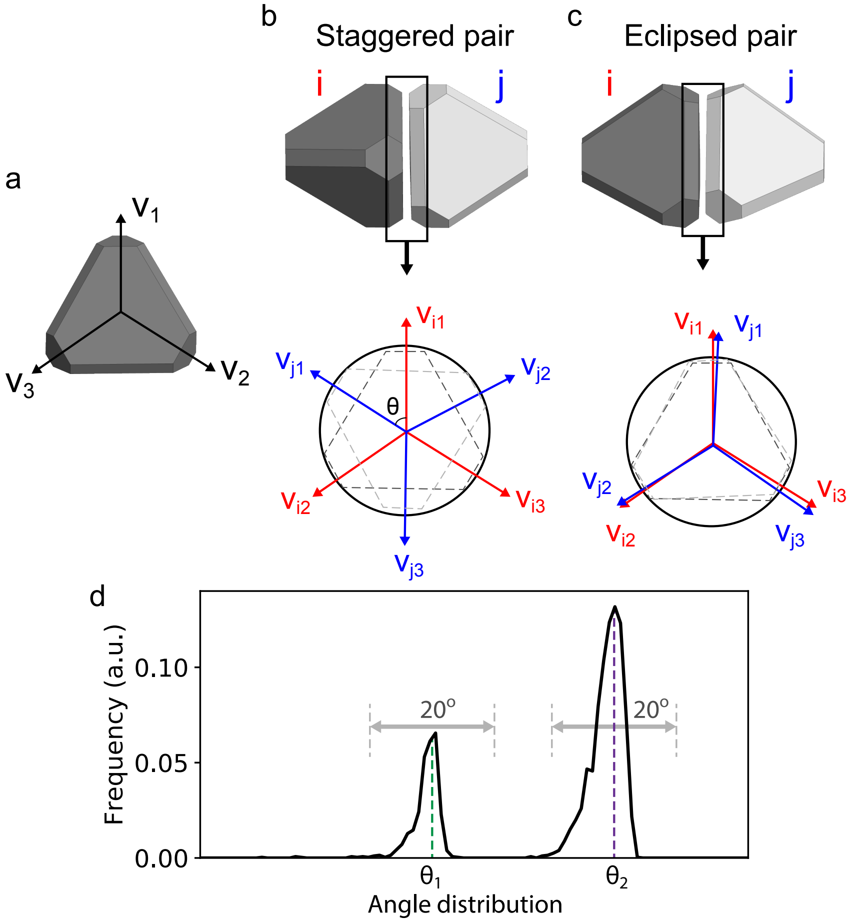

Supplementary Fig. 1. Pair contact type identification. **a**, Three vectors are assigned to each face of a particle. **b,c**, For each pair contact of i and j TTs (top), we found a minimum angle (θ) between the three vectors of i and the three vectors of j (bottom). **d**, Distribution plot of θ for a hexagonal diamond crystal.

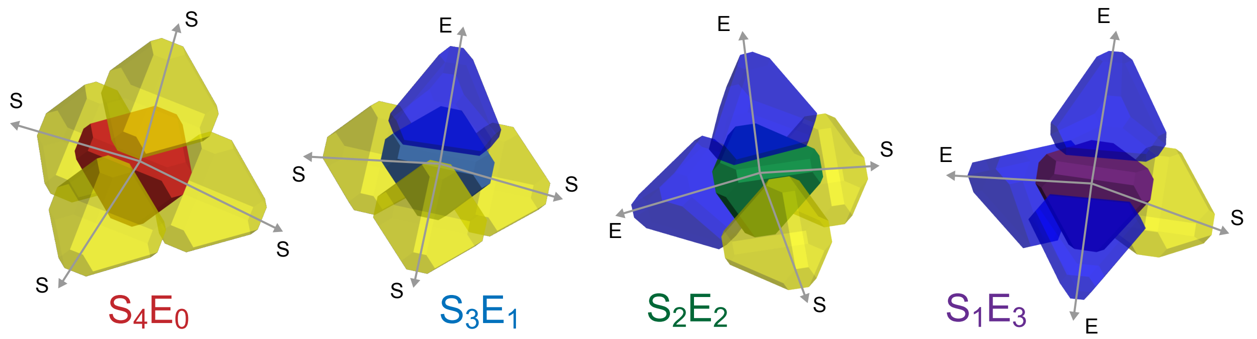

Supplementary Fig. 2. Four different local environments of a TT found in this study. S_4E_0 is found in the cubic diamond phase (Fig. 1b, 1g). S_3E_1 is found in the hexagonal diamond phase (Fig. 1c) and in twin planes (Fig. 1h). S_2E_2 is found at the center of the fivefold twinned cluster (Fig. 1i). S_1E_3 is found at the center of an icosahedral cluster, which forms a dodecahedron motif with 20 TTs (Fig. 1i, 1j).

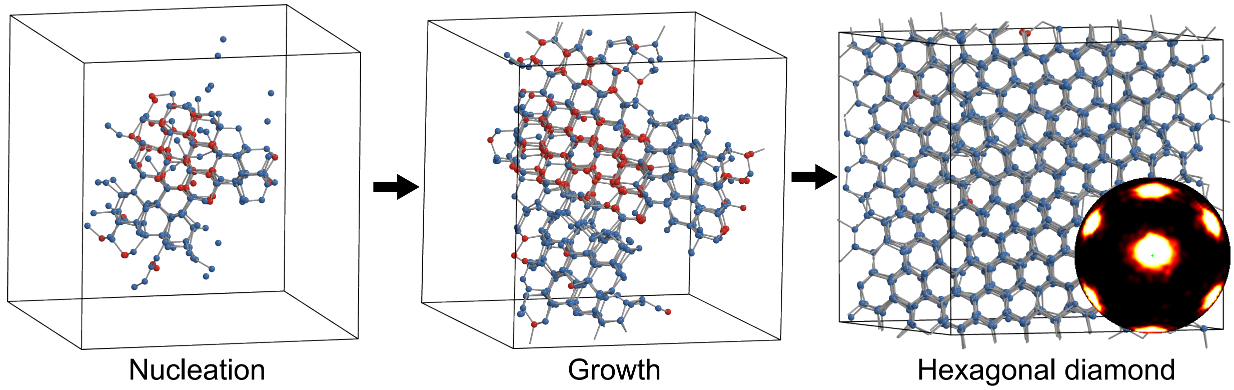

Supplementary Fig. 3. Self-assembly simulation result of hexagonal diamond of a = 1.28, c = 1.28 hard TT system with $N = 2000$ and $\phi = 0.63$, at constant volume. Simulation snapshots were obtained at 20×10^6, 22×10^6 and 48×10^6 MC steps.

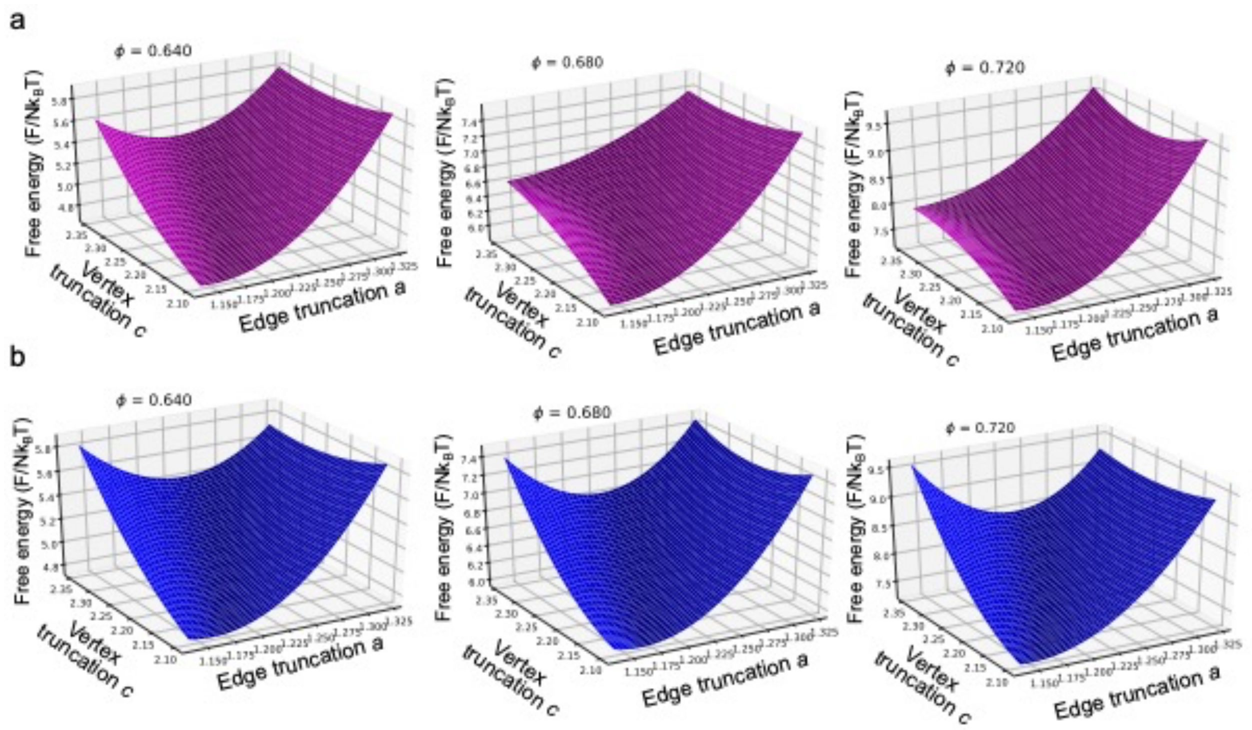

Supplementary Fig. 4. Per-particle free energy (F/Nk_BT) surface calculation results, in units of k_BT. **a,** Helmholtz free energy surfaces of the cubic diamond phase at three different particle volume fractions ($\phi = 0.64, 0.68$ and 0.72). b, Helmholtz free energy surfaces of the hexagonal diamond phase at three different particle volume fractions ($\phi = 0.64, 0.68$ and 0.72).

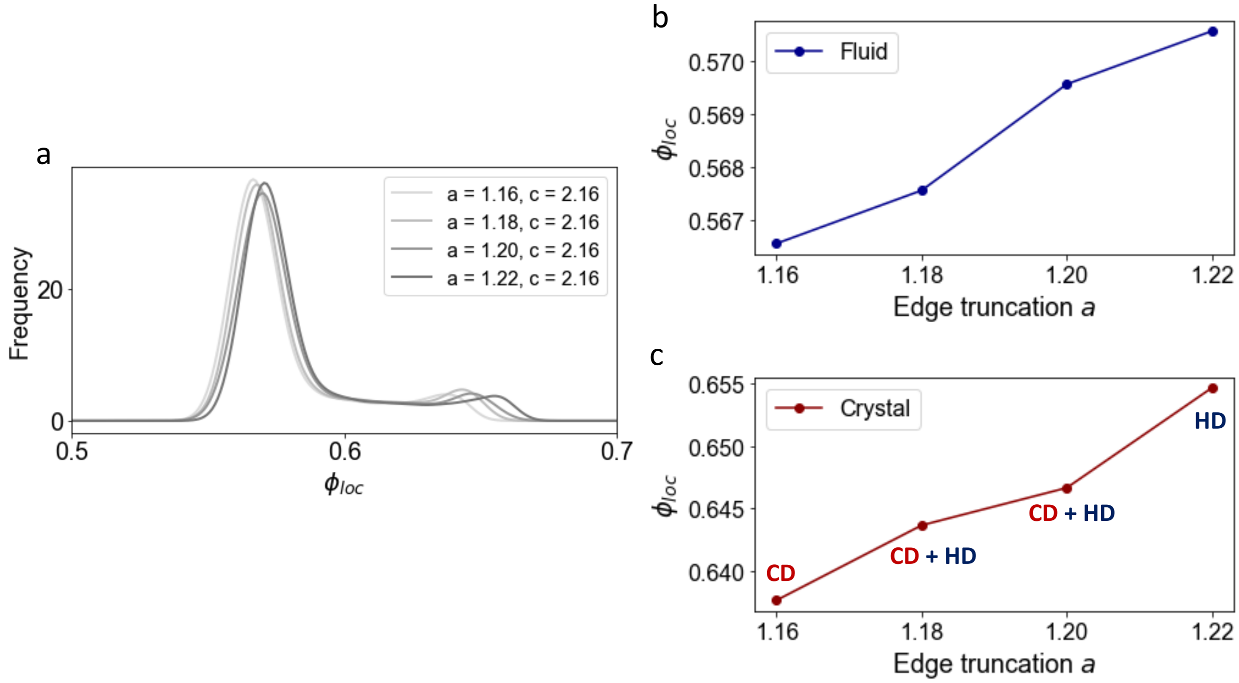

Supplementary Fig. 5. The dependence of local volume fraction on particle shape. (**a**) Local particle volume fraction distribution at fluid-solid coexistence in five different systems with different edge truncation parameter $a = 1.16, 1.18, 1.20$ and 1.22. (**b**) Particle volume fraction of fluid for each of the four systems. Each data point corresponds to the x-coordinate of the leftmost peak of each system in (a). (**c**) Particle volume fraction of crystal for each of the four systems. CD is cubic diamond and HD is hexagonal diamond. Each data point corresponds to the x-coordinate of the rightmost peak of each system in (a).

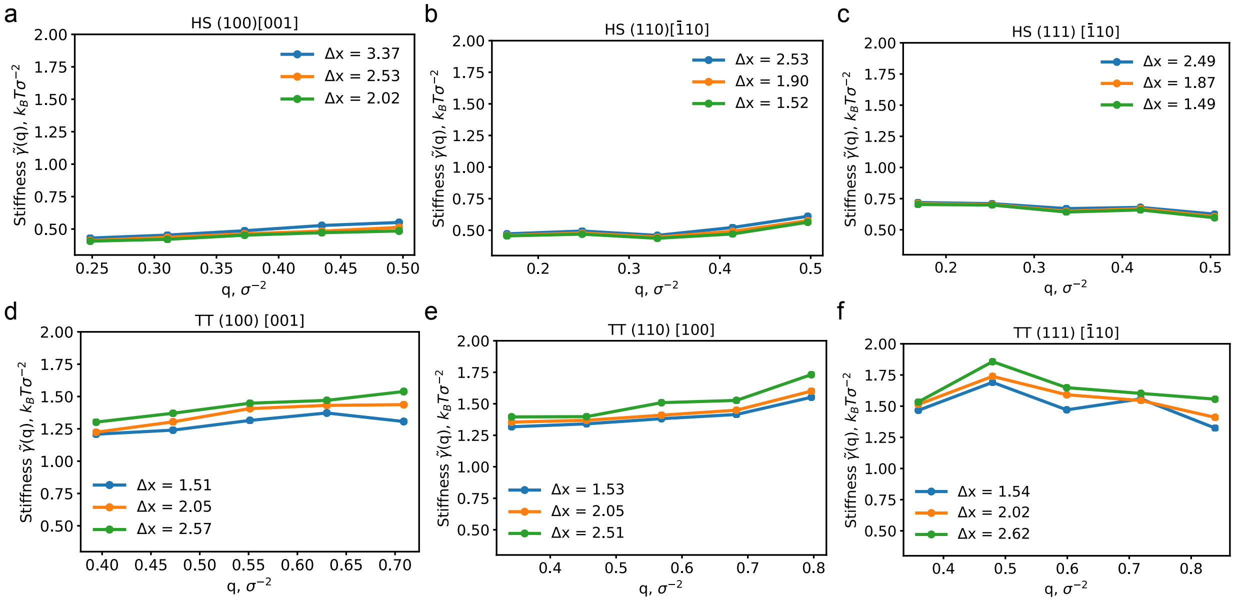

Supplementary Fig. 6. Fluid-solid interfacial stiffness of the fluctuation modes obtained from various parameters of the capillary fluctuation method. **a**-**c**, Stiffness of an FCC phase of hard spheres along three different orientations: (**a**) $(100)[001]$, (**b**) $(110)[\bar{1}10]$ and (**c**) $(111)[\bar{1}10]$. **d**-**f**, Stiffness of a cubic diamond phase of hard TTs along three different directions: (**d**) $(100)[001]$, (**e**) $(110)[100]$ and (**f**) $(111)[\bar{1}10]$. The results are averaged over 200 samples of the interfacial profiles obtained in equilibrium over 2×10^6 MC steps.

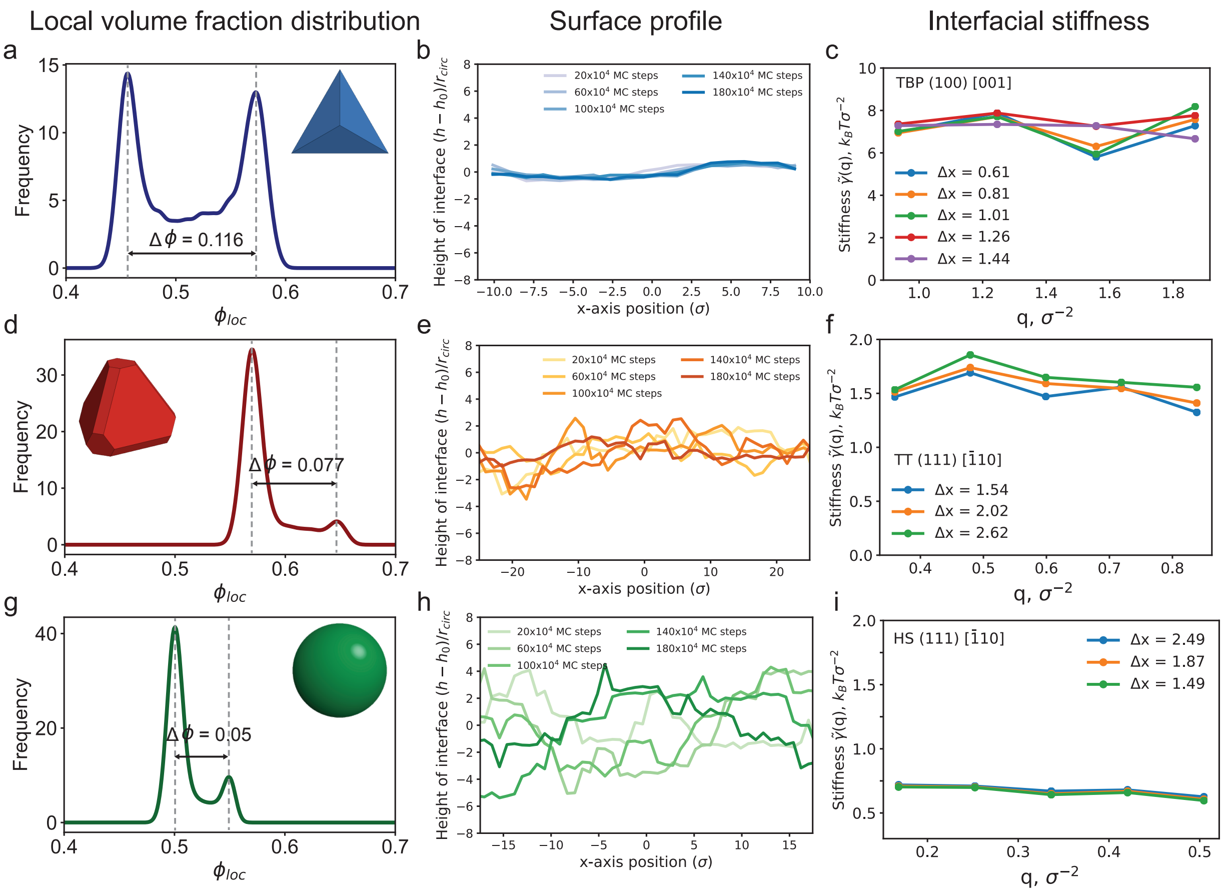

Supplementary Fig. 7. Fluid-solid interfacial tension depending on particle shape. (**a-c**) Clathrate type-1 of hard triangular bipyramids (TBPs). (**a**) Local volume fraction distribution at fluid-solid coexistence, and the local volume fraction difference between two phases is $\Delta\phi = \phi_{\text{Crystal}} - \phi_{\text{Fluid}} = 0.116$. (**b**) The change of interfacial profiles of the (100)[001] direction. (**c**) Stiffness of the interface. (**d-f**) Cubic diamond of hard TTs ($a = 1.20$ and $c = 2.16$). (**d**) Local volume fraction distribution at fluid-solid coexistence, and the local volume fraction difference between the two phases is $\Delta\phi = \phi_{\text{Crystal}} - \phi_{\text{Fluid}} = 0.077$. (**e**) The change of interfacial profiles of the $(111)[\bar{1}10]$ direction, which is the same plot to Fig. 4e. (**f**) Interfacial tension (the same plot in Supplementary Fig. 6f). (**g-i**) FCC of hard spheres (HS). (**g**) Local volume fraction distribution at fluid-solid coexistence. The local volume fraction difference between the two phases is $\Delta\phi = \phi_{\text{Crystal}} - \phi_{\text{Fluid}} = 0.05$. (**h**) The change of interfacial profiles of the $(111)[\bar{1}10]$ direction (the same plot as Fig. 4g). (**i**) Interfacial tension (the same plot as Supplementary Fig. 6c). The results are averaged over 200 samples of the interfacial profiles obtained in equilibrium over 2×10^6 MC steps.

Supplementary Table 1. A_1, A_2, of Eq. 3 and B_1, B_2 of Eq. 4 for each crystal plane. These values are same for both hard TT and hard sphere systems.

Interface orientation	A_1, A_2, B_1, B_2
$(111)[\bar{1}10]$	$-\frac{4}{15}, \frac{64}{63}, \frac{12}{5}, -\frac{1280}{63}$
$(100)[001]$	$\frac{2}{5}, \frac{4}{7}, -\frac{18}{5}, -\frac{80}{7}$
$(100)[\bar{1}10]$	$-\frac{1}{10}, -\frac{13}{14}, -\frac{21}{10}, \frac{365}{14}$
$(110)[001]$	$-\frac{1}{10}, -\frac{13}{14}, \frac{39}{10}, \frac{155}{14}$

Supplementary Table 2. Calculation results of γ_0, ϵ_1 and ϵ_2, and the interfacial free energy of hard TT and hard sphere system.

	Hard TT (Cubic diamond)	Hard spheres (FCC)
γ_0	1.3620	0.5717
ϵ_1	0.0306	0.0773
ϵ_2	-0.0031	-0.00053
γ_{100}	1.3763	0.5893
γ_{110}	1.3517	0.5676
γ_{111}	1.3467	0.5596
