# Supplementary material for: Entropically engineered formation of fivefold and icosahedral twinned clusters of colloidal shapes
Source: Nat Commun. 2022 Nov 30;13:7362. doi: 10.1038/s41467-022-34891-5 (PMC9712591; doi:10.1038/s41467-022-34891-5)
Supplement: Supplementary file 2 — Description of Additional Supplementary Files [file 41467_2022_34891_MOESM2_ESM.pdf]

Description of Additional Supplementary Files

Supplementary Movie 1. HPMC simulation of hard-TT ($a = 1.20, c = 2.16$) in a fluid-solid (cubic diamond) coexistence state ($\phi = 0.582$) for the capillary fluctuation method.

Supplementary Movie 2. HPMC simulation of hard spheres in a fluid-solid (fcc) coexistence state ($\phi = 0.515$) for the capillary fluctuation method.
